# The relationship between intestinal parasites and some immune-mediated intestinal conditions 

**Published:** 2015

**Authors:** Rasoul Mohammadi, Ahmad Hosseini-Safa, Mohammad Javad Ehsani Ardakani, Mohammad Rostami-Nejad

**Affiliations:** 1 *Department of Medical Parasitology and Mycology, School of Medicine, Isfahan University of Medical Sciences, Isfahan, Iran, *; 2 *Department of Medical Parasitology and Mycology, School of Public Health, Tehran University of Medical Sciences, Tehran, Iran*; 3 *Gastroenterology and Liver Diseases Research Center, Shahid Beheshti University of Medical Sciences, Tehran, Iran*

**Keywords:** Celiac disease, Inflammatory bowel diseases, Irritable bowel syndrome, Intestinal parasites

## Abstract

Over the last decades, the incidence of infestation by minor parasites has decreased in developed countries. Infectious agents can also suppress autoimmune and allergic disorders. Some investigations show that various protozoa and helminthes are connected with the main immune-mediated intestinal conditions including celiac disease (CD), inflammatory bowel diseases (IBD) and irritable bowel syndrome (IBS). Celiac disease is a digestive and autoimmune disorder that can damage the small intestine and characterized by a multitude gastrointestinal (GI) and extra GI symptoms. IBD (including ulcerative colitis and Crohn’s disease) is a group of inflammatory conditions of the small intestine and colon. The etiology of IBD is unknown, but it may be related to instability in the intestinal microflora that leading to an immoderate inflammatory response to commensal microbiota. Irritable bowel syndrome (IBS) is a common, long-term condition of the digestive system. Bloating, diarrhoea and/or constipation are nonspecific symptoms of IBS. Various studies have shown that some intestinal parasites can effect on immune system of infected hosts and in some cases, they are able to modify and change the host’s immune responses, particularly in autoimmune disorders like celiac disease and IBD. The main objective of this review is to investigate the relationship between intestinal parasites and different inflammatory bowel disorders.

## Introduction

**Figure 1:**
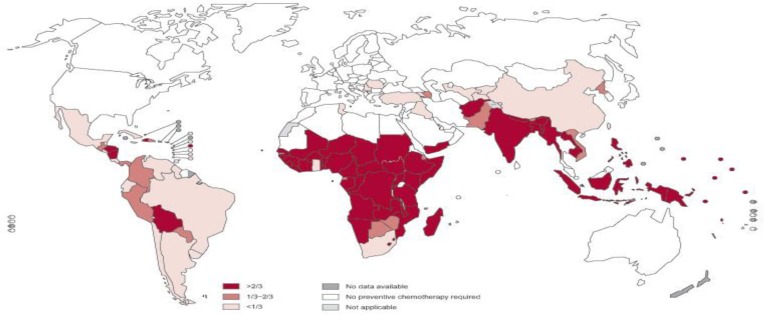
Global prevalence of Soil Transmitted Helminths (Source: World Health Organisation)

 Parasites and microbes have been important for adjusting and forming the human immune system (1). Industrialized countries are actually experiencing rising in some autoimmune disorders. Loss of parasite colonization in those individuals living in developed countries has had a remarkable impact on our immune response and it is likely the chief factor contributing to the progression of autoimmune diseases (2, 3). Figure 1 shows global prevalence of soil-transmitted helminths. Celiac disease (CD), inflammatory bowel diseases (IBD) and irritable bowel syndrome (IBS) are the most important immune-mediated intestinal conditions. Celiac disease is an autoimmune disease of the small intestine typically leading to malabsorption and affects many organ systems. It can involve people of all ages from middle infancy to old age (4, 5). When someone with celiac disease consumes gluten, his or her immune system assaults the lining of the small intestine. Gluten is a mixed protein composed mainly of the gliadin and glutenin that found in wheat, barley, and rye. Consuming gluten-containing foods can initiate a range of gastrointestinal symptoms like abdominal pain, diarrhea, flatulence, bloating, weight loss, and extra intestinal signs such as anemia, osteoporosis, infertility and nervous problems (6).

**Figure 2:**
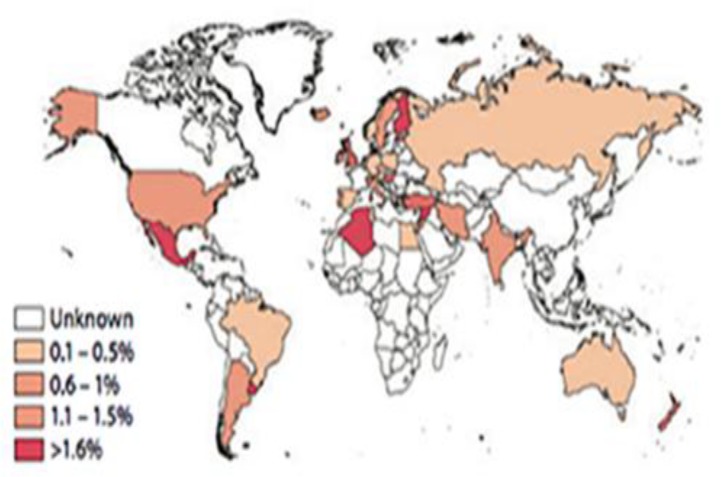
Global prevalence of celiac disease (Source: Annual Review of Immunology Vol. 29)

**Figure 3:**
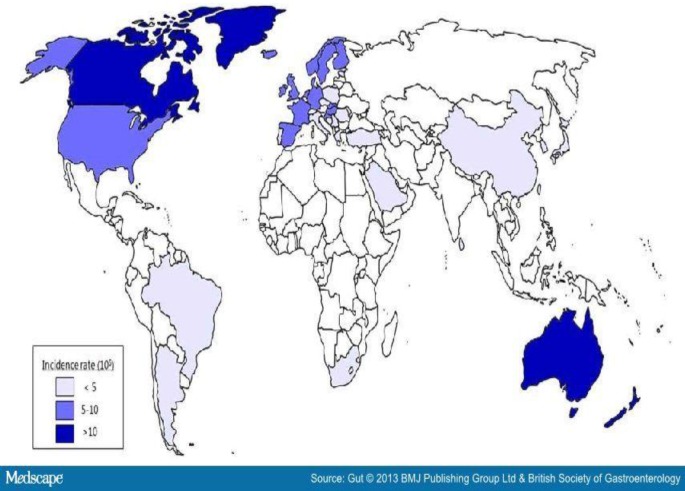
Global prevalence of Inflammatory Bowel Disease (Source: BMJ Publishing Ltd & British Society of Gastroenterology).

The only treatment for CD is a long life gluten free diet. Figure 2. shows the global prevalence of celiac disease.

**Figure 4:**
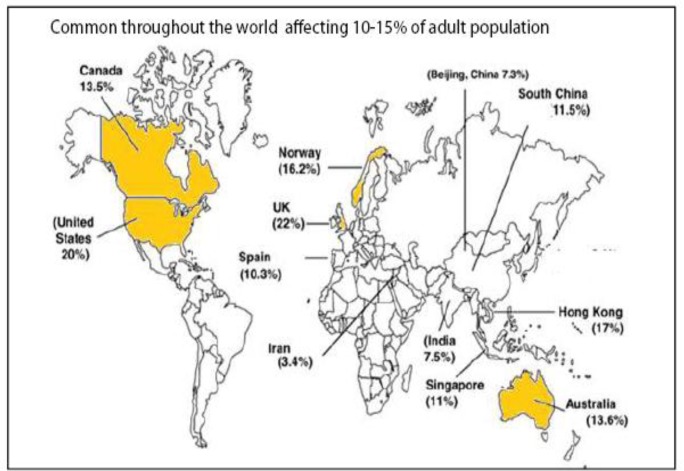
Worldwide incidence of IBS

IBD is an idiopathic, chronic, and recurring inflammatory disease of the gastrointestinal tract, which is represented principally by ulcerative colitis (UC) and Crohn's disease. Lately, the intestinal microbiota have been considered to be a significant factor in their etiology (7). UC is a worldwide chronic inflammatory disorder of the colon that causes typical ulcers in the mucosa of the rectum and colon (8). On the other hand, Crohn's disease is a chronic inflammatory condition that can influence any part of the gut from mouth to anus (9). Methylated thiopurine metabolites, like 6-methyl mercaptopurine, are frequently used for the treatment of IBD (10). Global prevalence of Inflammatory Bowel Disease has been shown in figure 3. IBS is a gastrointestinal disorder typically present with chronic abdominal pain and changed bowel habits (11). Recent investigation presented that IBS is characterized by meaningful alterations in the gut microflora (12). Many studies have shown that gastrointestinal infection is an important risk factor for the development of IBS (13, 14). IBS prevalence varied according to diagnostic criteria and geographic regions (figure 4). 

A parasite is an organism that takes its food from another organism. Parasitic diseases comprising infections that are caused by protozoa, helminths or arthropods. Some studies have shown that many parasites such as hookworm can induce not only parasite-specific immunity, but also they modified the host’s immune responses (15-17). Many parasites can imitate inflammatory bowel disorders (18) and some studies showed that infection with helminthes can improve disorders like IBD or moderate the symptoms of inflammatory bowel disorders (19). This review was focused on correlation between intestinal parasites and inflammatory bowel disorders. 

## Celiac disease

***Hookworms and Immune system***

Investigations in animal models revealed strong evidence that helminths can downregulate parasite-specific immune responses adjust autoimmune responses and enhance metabolic homoeostasis (20). Gaze, et al. (21) and Croese, et al. (22) showed experimental hookworm infection cause robust mucosal Th2, Th1, and regulatory responses, and upregulates IL-15 and ALDH1A2 (a complex recognized to elevate Th17 inflammation in celiac disease) in celiac disease patients. During hookworm infection, suppression of mucosal IL-23 and also upregulation of IL-22 was occurred. Accordingly, Th17 responses are suppressed and inflammatory in celiac disease are blocked. Contrary to both celiac and Crohn’s diseases, hookworm infection suppressed in circulating CD4+CD25+Foxp3+cells (23, 24). 

***Necator americanus***

In the celiac disease, the responses to gluten are considerably different if *N. americanus* was present. Dermal immunization of *N. americanus* has also been used to regulate the immune response to gluten (25). Therefore, hookworm infection can decrease gluten sensitivity and can use to treat celiac disease.

***Treatment***

In many countries the patients intentionally infect with worms and this is considered as a possible treatment for inflammatory diseases, for example, use of the *Necator americanus* larvae for treatment of celiac disease (20). Long-term use of low-dose *N. americanus* seems to be safe in CD treatment (22), due to the changing of immune responses such as cytokine production like IL-1β, IL-15 and IL-22 (26). Slack described that extended infectious diarrhea in the visitors is usually caused by protozoal and helminth parasites, and disease symptoms/treatment should be considered from non-infectious diarrhea such as celiac disease (27). 

***Other parasites***

Jiménez et al. (28) reported that *Blastocystis hominis* should be noted as an opportunistic pathogen in characteristic celiac patients with low weight and subtotal-total villous atrophy. Celiac disease and *Giardia intestinalis* are usual causes of dyspepsia. Fouad, et al. (29) showed that *G. intestinalis* genotype A demonstrated a greater connection with dyspepsia. In both children and adults, untreated celiac disease is the most common cause of malabsorption syndrome, and in these patients some pathogenic parasites like *Giardia lamblia, Ancylostoma duodenale, Entamoeba histolytica/dispar, Cyclospora cayetanensis, Hymenolepis nana, Cryptosporidium, Cyclospora *and* Isospora belli* more often colonized compared to healthy control (30). Rostami Nejad, et al. (31) reported that *Toxoplasma gondii* infection rate was higher among patients with CD-serology positive than among patients with negative CD serology. Lidar, et al. (32) showed that raised titers of *T. gondii* antibodies have been observed in celiac disease patients and individuals with inflammatory bowel disease. 

## Inflammatory bowel disease (IBD)

***Strongyloides stercoralis***

Many investigations showed the curative effects of a controlled parasitic nematode infection on autoimmune disorders like IBD (33-35), but the exact mechanism(s) of these effects are not clear. *Strongyloides stercoralis*, an intestinal nematode, is common intestinal parasite in tropical areas e.g. Southeast Asia, Latin America, Sub-Saharan Africa, and many European countries (36). As it can occasionally imitate IBD, particularly UC, it is important to be kept out (18).

***Blastocystis***

Cekin, et al. (37) reported that Blastocystosis was more frequent in patients with IBD, specifically those with UC, but no statistically differences were shown between IBD patients and control group. 

***Toxoplasma gondii***

Macrophage migration inhibitory factor (MIF) is an important mediator for controlling parasitic infections such as *T. gondii* (38-40). Cavalcanti, et al. (41) showed that *T. gondii* infection influences small intestine necrosis and death in sensitive patients with inflammatory bowel disease. They suggested that MIF contributed to the inflammatory response caused by oral infection with *T. gondii.*

***Helminths***

The result of clinical trial showed that infection with helminthes can improve IBD (19). On the other hand, an increasing prevalence of IBD in western countries could be associated whith the lower prevalence of intestinal helminthes (42). Moreels, et al. (43) illustrated that *Trichuris suis* can moderate IBD symptoms. Weerasekara, et al. (44) reported that patients with mild IBD symptoms might have been exposed to helminths like *N. americanus, Trichuris trichiura, Ascaris lumbrcoides *and* Enterobius vermicularis*, in childhood. Elliott, et al. (45) showed that helminths colonization change Th2 and regulatory immune responses such as IL-4, IL-5, IL-10, and IL-13 production, and save animals from progressing immune-mediated diseases like ulcerative colitis or Crohn's disease. Some evidences demonstrated that rodent nematodes like *Trichuris muris, Nippostrongylus brasiliensis*, and *Trichinella spiralis*, shift the gastrointestinal immune status toward Th2 production (43, 46). Motomura, et al. (47) revealed that previous treatment with *T. spiralis* antigens in mice decreased the intensity of colitis and the mortality rate significantly. They explicated that up-regulation of transforming growth factor-β (TGF- β) and IL-13 and down-regulation of interleukin (IL)-1β production, myeloperoxidase (MPO) activity, and nitric oxide synthase (iNOS) expression in colon are related to decreasing disease mortality. Khan, et al. (48) showed that *Trichinella spiralis* protected mice from colitis and IBD. Moreover, schistosome eggs provide a defensive effect on TNBS-induced colitis in mice (49). Infection with *Heligomosomoides polygyrus* or *Trichuris muris* can prevent or invert the chronic Th1-type colitis in IL-10 deficient mice (50). Helminths and their products can modulate the innate and adaptive immune system (51). Summers, et al. (52, 53) showed *T. suis* is well tolerated and appears effective for Crohn’s and ulcerative colitis. 

## Irritable bowel syndrome (IBS)

Various parasites comprising *B. hominis, Giardia spp., E. histolytica*,* Dientamoeba fragilis* and* Trichinella spp.* have been considered as contributing factors to the progress of IBS (54-56), although the correlation is not confirmed. 

***Blastocystis***

 Dugroman, et al. (54) reported that *Blastocystis* infection was found in 67% of the patients with IBS and it could be a serious problem for diagnosis of IBS. In a comprehensive study was performed on 357 parasite carriage among IBS cases in Nicaragua, Morgan, et al. reported that statistically difference was not noticed in the prevalence of intestinal parasite infection including *B. hominis, G. lamblia, E. histolytica/dispar, E. nana, A. lumbricoides, *and* H. nana* among patients with IBS compared to the healthy controls (57). 

***Giardia*** **Spp.**

Dizdar, et al. (58) found that post-infectious bowel dysfunction following *Giardia* infection is associated with increased duodenal mucosal in IBS patients. 

***Dientamoeba fragilis***

The first time, Borody, et al. reported the relationship between IBS and *Dientamoeba fragilis *(59). Epidemiological studies have shown the range of 2–4% of *D. fragilis* in IBS patients (60, 61). Among 25 *D. fragilis*-positive IBS patients, Engsbro, et al. (62) found no relationship between *D. fragilis* and IBS. *D. fragilis* can cause IBS-like symptoms. Therefore, it is not unexpected that many patients with *D. fragilis* are misdiagnosed as having IBS. 

***Haplorchis taichui***

*H. taichui* is a fluke that belongs to the family Heterophyidae. It can live in the small intestines of mammals and birds and is found in Southeast Asia (63). Humans become infected with eating the metacercariae in infected cyprinoid fish (64). Watthanakulpanich, et al. (65) showed that *H. taichui* could be a possible etiologic agent for IBS-like symptoms. 

***Trichinella***

For the first time, Soyturk, et al. (66) declared that IBS could be considered as a secondary syndrome caused by trichinellosis.

***Trichuris trichiura***

 Diniz-Santos, et al. (67) showed that *T. trichiura* could be misdiagnosed due to its ability to mimic IBS symptoms. Table 1 summarizes some examples that show the relationship between intestinal parasites and inflammatory bowel disorders.

**Table 1:** The association between intestinal parasites and celiac disease, inflammatory bowel disease and inflammatory bowel syndrome

	Celiac disease	IBD	IBS
*N. americanus*	Down regulation of immune response to gluten	-	-
*B. hominis*	Opportunistic pathogen in CD patients with low weight and subtotal-total villous atrophy	-	-
*Giardia *spp.	It is usual causes of malabsorption and dyspepsia	-	-
*T. gondii*	-	Influences small intestine necrosis	-
*S. stercoralis*	-	Imitate IBD symptoms	-
*T. suis*	-	Can moderate IBD symptoms	-
*T. spiralis*	-	protected mice from colitis and IBD	-
*D. fragilis*	-	-	It is considered as a contributing factor to the development of IBS, can cause IBS-like symptoms
*H. taichui*	-	-	It can be a possible etiologic agent for IBS-like symptoms

## Conclusion

In many countries deliberate infection with helminthes larva considered as a possible treatment for inflammatory disorders like celiac disease due to the changing of immune responses such as cytokine production. Various surveys showed the therapeutic effects of a controlled parasitic infection on autoimmune disorders. Some studies reported that hookworm infection decrease gluten sensitivity and can employ to treat celiac disease. It is suggested that rising prevalence of inflammatory bowel disorders such as IBD could be associated with the decreased prevalence of intestinal helminthes. This review has highlighted some of these assumptions. However, the precise mechanisms of these effects remain unclear. According to these studies we think that there is a relationship between some parasitic infections such as Toxoplasma gondii, and the development of CD, helminthes infections and development of IBD, Dientamoeba fragilis and B. hominis and development of IBS. Understanding the correlation between parasitic infections and autoimmune disorders may be helpful in prediction, early identification and conceivably the prevention of these diseases.
